# Working Memory Updating Function Training Influenced Brain Activity

**DOI:** 10.1371/journal.pone.0071063

**Published:** 2013-08-27

**Authors:** Xin Zhao, Renlai Zhou, Li Fu

**Affiliations:** 1 Beijing Key Laboratory of Applied Experimental Psychology, School of Psychology, Beijing Normal University, Beijing, China; 2 School of Psychology, Northwest Normal University, Lanzhou, China; University Of Cambridge, United Kingdom

## Abstract

Recent studies demonstrated that working memory could be improved by training. We recruited healthy adult participants and used adaptive running working memory training tasks with a double-blind design, combined with the event-related potentials (ERPs) approach, to explore the influence of updating function training on brain activity. Participants in the training group underwent training for 20 days. Compared with the control group, the training group's accuracy (ACC) in the two-back working memory task had no significant differences after training, but reaction time (RT) was reduced significantly. Besides, the amplitudes of N160 and P300 increased significantly whereas that of P200 decreased significantly. The results suggest that training could have improved the participants' capacity on both inhibitory and updating.

## Introduction

Working memory is the ability to maintain and manipulate information temporarily while an individual performs cognitive tasks [1], [2], [3], [4], it is the core of human high-level cognitive activities and an essential component in the processes of learning, reasoning, problem solving and intelligence [5], [6], [7], [8], [9], [10], [11], [12]. In recent years, training results from children [13], [14],adults [15], [16],and people with attention deficit/hyperactivity disorder [17],alcohol spectrum disorders[18] and stroke [19] have demonstrated that an individual's working memory ability is plastic. Several researchers have used neuroimaging techniques to explore the neural basis of working memory plasticity. Their results showed that, through working memory training, the activity of the brain areas related with working memory can be enhanced [16], [20]. McNab et al. found that the plasticity of working memory is correlated with changes in the density of the dopamine D1 receptor in the cerebral cortex [21].

Although these cognitive neuroscience studies proved that working memory training could change the activity of relevant brain areas [16], [20], the brain areas involved in working memory are complicated, and a certain connectivity of those different areas may be required to fulfill this function [22]. However, results from neuroimaging techniques show that it is difficult to explain the influence of working memory training on brain activity based on the spatial activation pattern. The event-related potentials (ERPs) technique has high resolution in a time course and could distinguish the changes in different stages of working memory processing based on the temporal activation patterns. For instance, McEvoy et al. found that the aging of working memory is not a single-area alteration [23]. On the contrary, aging was revealed by decreases in the amplitude of P300 in the parietal cortex and increases in the amplitude of P200 in the frontal cortex. Thus, our study used ERPs to probe the influence of working memory updating training on the relevant brain processing.

Updating is an essential component in the central executive component of WM, which recently have attracted great attention in the WM study field [14], [24]. The major function of updating is to continuously and simultaneously change the contents in the working memory load according to newly presented information [22], [25]. The running memory task is widely used as an index of WMU. In this task, participants are presented with a series of unknown items of a certain length, which they are required to recall in order within a certain length of time. This task better represents the ability to monitor input information and to replace old information that is irrelevant to the ongoing task with new information that is relevant to the ongoing task [26]. More important, the running memory task purely reflects the central executive functioning involved in WM, removing the potential confounding effects of storage. Our studies used an adaptive running working memory task to perform working memory updating training.

## Methods

### Ethics Statement

The experiment was conducted after obtaining Institutional Review Board approval from the School of Psychology at Beijing Normal University. All participants gave informed written consent before testing began.

### Participants

A total of 24 right-handed adult participants in good spirits were recruited to participate in the experiment. The participants were between 18 and 29 years of age, with normal or corrected normal sight, no color blindness or weakness, no history of mental illness and neuropathy, no medical treatment or drug use before the experiments, and no consumption of coffee, alcohol or other substances that could affect the neurosystem 24 hours before the pre- and post-test. The subjects were randomly divided into training and control groups, with 12 subjects in the training group (3 males and 9 females with an average age of 20.5 years, std 2.24) and 12 subjects in the control group. However, the cap of one participant in the latter group was off and did not join in the post-test, so there were 11 valid participants (5 males and 6 females with an average age of 21.27 years, std 3.10). The experiments were approved by the IRB in Beijing Normal University, and all of the participants signed consent forms and received ordinary pay.

### Tests for Pre- and Post-training Evaluation

We used a 2-back task to measure working memory ability, which has a sound validation and reliability; the program was compiled by E-prime 2.0. In the 2-back task, the participants were asked to compare whether the current stimulus is the same as the latest two stimuli. When performing the task, there was a fixation “+” in the center of a black screen, and a series of Arabic numbers would present up/down/left/right of the “+” one by one; the numbers range from 1 to 9. The participants need to judge the numbers' locations regardless of their values. The font used was “Times New Roman”, the color was white, the font size was 60, and the numbers and the positions that they appeared in were random. The interval between stimuli was 4500 ms: first the prompt “X” was presented, followed by a 1300 ms delay, and then the stimuli were presented for 200 ms, allowing participants to enter a reaction within 2500 ms. The whole test consists of 2 blocks with 84 trials, and the first two trials in each block need no response. Half of the participants press “1” for a consistent judgment and “3” for an inconsistent judgment, and the other half are press “3” for a consistent judgment and “1” for an inconsistent judgment. In every task, the ratio of consistent to inconsistent judgments is 1∶1.

### Computerized Training Program

#### Letter Running Working Memory Task

First, a fixation “+” was presented in the center of the screen to indicate the onset of the task. Several letters were then presented one after another. The number of letters presented varied between different trials, with 5, 7, 9 or 11 letters presented in each trial. Every trial type occurred in a random order. The participants were required to sequentially remember the last three letters presented. For example, if the presented letters are, sequentially, S-D-F-G-H-J-K, then participants need to remember S, S-D, S-D-F, D-F-G, F-G-H, G-H-J, H-J-K. Finally, a blank is presented on the screen, and the participants need to enter the last three letters presented, in order, using the keyboard. The duration of every letter presented is 1750 ms at the beginning, and the difficulty of the task is subsequently changed according to the scores of participants; namely, as their scores increase, the duration of every letter presented decreases. The participants finished 6 blocks with 5 trials of this task. To be specific, if participants correctly answered 3 or more trials in these five trials, the duration of each letter in the next block will be reduced by 100 ms. In contrast, if the participants incorrectly answered 2 or more trials in these five trials, the duration of each letter will be prolonged by 100 ms. The next day's training is based on the former day's record.

#### Animal Running Working Memory Task

First, a fixation “+” was presented in the center of the screen to indicate the onset of the task. Several images of animals were then presented one after another. The number of animal stimuli presented varied between different trials, with 5, 7, 9 or 11 images presented in each trial. Every trial type occurred in a random order. Participants were required to sequentially remember the last three animals presented. For example, if the presented animals are, sequentially, panda-dolphin-ostrich-goat-crocodile-dinosaur-rooster, then participants need to remember panda, panda-dolphin, panda-dolphin-ostrich, dolphin-ostrich-goat, ostrich-goat-crocodile, goat-crocodile-dinosaur, and crocodile-dinosaur-rooster. Finally, nine animals are presented on the screen at the same time, and participants need to press the last three animals presented, in order, with the mouse. The duration of every animal presented is 1750 ms at the beginning, and subsequently, the difficulty of the task is changed according to the scores of participants; namely, as their scores increase, the duration of every animal presented decreases. The participants finished 6 blocks with 5 trials of this task. To be specific, if the participants correctly answered 3 or more of these five trials, the duration of each animal in the next block will be reduced by 100 ms. In contrast, if the participants incorrectly answered 2 or more trials in these five trials, the duration of each animal will be prolonged by 100 ms. The next day's training is based on the former day's record.

#### Location Running Working Memory Task

**Figure 1:**
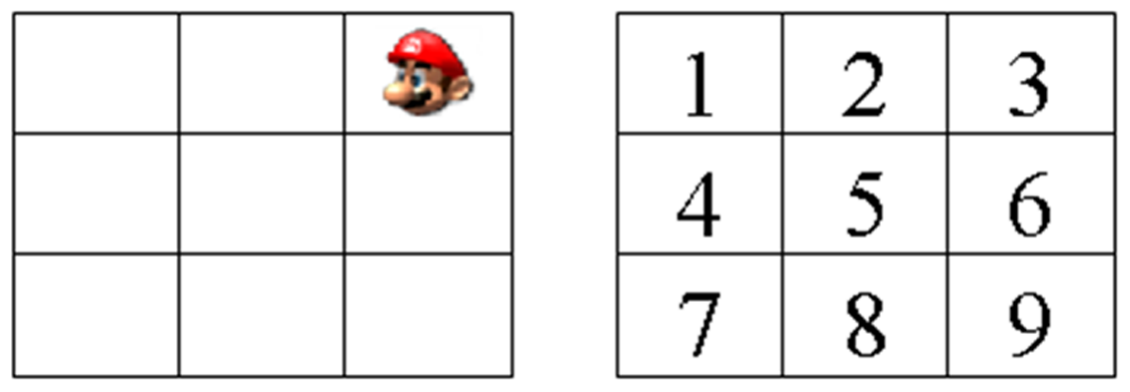
Demonstration of Location Working Memory Task.

Cartoon location running memory task: First, a fixation “+” was presented in the center of the screen to indicate task onset. A nine-square grid was then presented in the center of the screen, containing an image of a cartoon face (the Nintendo character “Mario”), which could occur in any of the nine squares (See Figure 1).The number of cartoon face presented varied among different trials, with 5, 7, 9 or 11 cartoon faces presented in each trial. Every trial type occurred in a random order. The participants were required to sequentially remember the last three locations of Mario presented. For example, if the presented locations of Mario were sequentially 2-7-5-3-1-4-9, then participants needed to memorize the location sequentially as 2, 2-7, 2-7-5, 7-5-3, 5-3-1, 3-1-4, 1-4-9 (See Figure 1). Finally, three grids were presented on the screen at the same time, and the participants needed to press the last three locations of Mario presented in order into the three grids with the mouse. The duration of every location of Mario presented is 1750 ms at the beginning, and subsequently, the difficulty of the task is changed according to the scores of participants; namely, as their scores increase, the duration of every location of Mario presented decreases. The participants finished 6 blocks with 5 trials of this task. To be specific, if participants correctly answered 3 or more of these five trials, the duration of each location of Mario in the next block will be reduced by 100 ms. In contrast, if participants incorrectly answered 2 or more of these five trials, the duration of each location of Mario will be prolonged by 100 ms. The next day's training is based on the former day's record.

After finishing one trial, participants would confirm their selection by clicking “sure” to begin the next trial. Correct responses were recorded as a score of “1”, meaning that the maximum total score was 30. When the answer was correct, a smiley-face figure was presented at the bottom of the screen as feedback. When the response was incorrect, a bomb figure appeared in the same location. The feedback was presented during every trial, and participants received rewards according to their scores.

In the training process, participants need to finish all the three computerized training programs once a day, with whatever sequence they would like. The time interval between pre- and post-test in both groups was 21–23 days. Pre-training test was conducted 1–3 days before the start of training process and post-test was conducted in 1–3 days after the training process.

### ERPs Data Collection and Offline Data Analysis

The electroencephalogram (EEG) was recorded by 64 Ag/AgCl electrodes mounted on a custom-made cap (ECI; Eaton, Ohio) according to the extended 10–20 system and continuously sampled at 1000 Hz by a Neuroscan Synamp2 Amplifier. The band-pass filter range of 0.05 to 100 Hz was used during the EEG recording. Vertical EOG and horizontal EOG were recorded with two pairs of electrodes, one placed above and below the right eyes and another 10 mm from the lateral canthi. Both the EEG and EOG were referenced on-line to the left mastoid and re-referenced off-line with the average of the bi-mastoids. Throughout the EEG recording, the impedance of the electrodes was maintained under 5 kΩ.

Remaining artifacts exceeding ±100 µV in amplitude or containing a change of over 100 µV within a period of 50 ms were rejected. The artifact-free EEG was then segmented into epochs ranging from 200 ms before to 800 ms after stimulus onset and averaged separately for each participant and for each condition. Only correct responses were included in the average, but all averages included at least 50 trials. The averaged waveforms were digitally low-pass filtered at 30 Hz (24 dB/octave) to reduce high-frequency noise.

## Results and Discussion

### Behavioral Results

The additive values were calculated by the post- minus pre-training RT and ACC of the 2-back working memory task (See Table 1). The training and control groups were calculated separately. The results of the independent sample t-test of the two additive values demonstrated that there is no statistically significant difference related to the additive ACC values between the training and control groups (*t*(21) = 1.243, *p* = 0.228, *d* = 0.52). The additive RT values of the training group were lower than those of the control group (*t*(21) = −2.405, *p* = 0.025, *d* = 1.00). We didn't find an increase of accuracy in 2-back task in post-test, compared with pre-test, which might be due to the difficulty of the task and the cognitive capacity of our participants. The 2-back task adopted in our study had been verified to have good validity, reliability, and differentiation [27]. Whereas, in our study, all participants are aged 18–29, and all are well educated. Accuracy of 23 participants ranges from 77.5% to 100%, and mean accuracy of the pre-test was 95.27%, and the standard deviation was 5.01. As it demonstrated in Table 2, one participant's accuracy was 77.5%, one 88.5%, one 89.7%, two 92.4%, two 93.7%, one 94.8%, one 94.9%, one 96.2%, two 96.3%, one 97.4%, three 97.5%, two 98.7%, two 98.8%, and three 100%. Judging from the overall data distribution, it can be found that accuracy for pre-test was quite high, with most of them around 95%, which might leave little room for participants to improve. However, it reflects at the same time that the participants had fully understood the task and thoroughly devoted their cognitive resources to accomplish the task. Analyzing data of each participant in the training group, we found that 8 out of 12 improved in accuracy, 2 subjects showed no difference, 2 declined. See Table 2.

**Table 1:** Accuracy (ACC; %) and Reaction Time (RT; ms) of pre and post training 2-back working memory task.

Group	Index	Pre Test	Post Test	Additive Value
Training Group(n = 12)	ACC	93.15±5.83	94.08±3.53	0.93±4.65
	RT	1005.63±266.70	760.93±190.83	−244.70±200.92
Control Group(n = 11)	ACC	97.59±2.55	96.15±4.12	−1.44±4.52
	RT	939.94±218.53	866.09±191.90	−73.85±128.20

Excluded data outside three standard deviations (STD) and wrong responses.

**Table 2:** Accuracy of pre-test and post-test in working memory 2-back task for 12 subjects in training group.

Subject	Accuracy of Pre-test	Accuracy of Post-test	Improve or not
Sub1	88.5	93.7	Y
Sub2	96.2	96.2	-
Sub3	94.8	97.5	Y
Sub4	96.3	98.7	Y
Sub5	97.5	91.1	N
Sub6	89.7	89.9	Y
Sub7	93.7	94.9	Y
Sub8	77.5	87.3	Y
Sub9	98.8	91.0	N
Sub10	97.5	97.5	-
Sub11	94.9	96.2	Y
Sub12	92.4	95.0	Y

While no significant improvements on accuracy were shown in post-test over the pre-test, the reaction time for working memory 2-back task decreased significantly. Our explanations are: a) No trade-off effects exist between accuracy and reaction time in the pre- and post-test; b) Evidence from previous studies [23], [27], [28] showed that reaction time of working memory 2-back task is an indicator of working memory capacity. McEvoy found the reaction time of old people in working memory 2-back task is significantly higher than that of the younger ones, thus making the reaction time a main indicator of working memory aging [23]. Gevins found the subjects with higher IQ showed a significant lower reaction time in working memory 2-back task, making the reaction time a main indicator of intelligence level [28]. Therefore, we regarded reaction time as an effective indicator of evaluating the training effect.

Our study used the running memory task, which purely reflects the central executive functioning involved in WM and removes the potential confounding effects of storage from the training task. Compared with the control group, the training group's 2-back working memory task performance improved significantly, as demonstrated by the decrease in the RT. The 2-back task involves storage capacity and multiple executive sub-components [27]. The improvement in 2-back working memory performance resulted from an improvement in executive function, namely, the core updating function in working memory.

### ERPs Analysis

Following previous research [23], for our total average results, we mainly measured P200 in the frontal area (FZ) and P300 and N160 in the parietal area (PZ, P7, and P8). The amplitudes of P200 and N160 were apparent; hence, we measured their peak values. The time window of P200 was 150–250 ms and that of N160 was 130–200 ms. The peak value of P300 had large individual differences, and several participants did not have apparent peak values. Therefore, we merely analyzed the average amplitude, and we chose 250–500 ms as the time window; this decision was based on previous research and the additive results.

**Figure 2:**
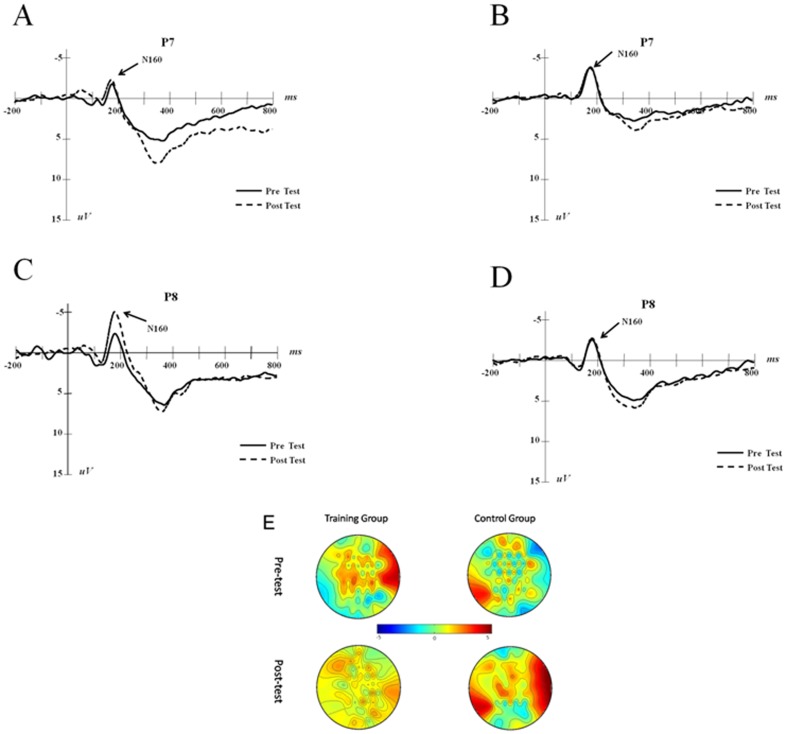
N160 trends of training and control groups (P7, P8). A and C are the training group results of pre- and post-test, and B and D are the control group results. Scalp topographies of the two groups on N160 are demonstrated on E.

For the individual N160 component analysis, we used the post-test peak value minus the pre-test peak value to obtain the additive values, and we calculated the two groups' average additive values (See Figure 2). The independent-sample t test showed that, on the P7 electrode, the additive value of N160 is not significantly different between the two groups (*t*(21) = −0.735, *p* = 0.470, *d* = 0.30) whereas, on the P8 electrode, the training group's N160 additive value is significantly lower than the control's (*t*(21) = −2.356, *p* = 0.028, *d* = 0.98). These results showed that the N160 amplitude increased after training. The N160 component is considered to be representative of visual recognition processing [23]. The increased N160 amplitude after training demonstrated that working memory updating training can improve the strength and validity of an individual's recognition of a target stimulus. This result implied that the influence of training likely occurs at the perception stage. In our study, the positive results on P8 combined with the negative results on P7 may be due to the spatial task used in the pre- and post-tests. The N160 component was found in the studies of facial identity earlier. N160 (N170) was recorded during 130–200 ms after the appearance of face or other objects, whose negative amplitude arrives during 160–170 ms. It mainly shows in occipito-temporal region and gets the highest amplitude on P8 (T6).When induced by face, the N160 (N170)'s amplitude gets higher than that induced by objects on both hemisphere, usually with right hemisphere advantage [29]. Previous studies revealed that N160 (N170) might reflect the process of stimulus's category in recognizing progress. N160 (N170) could be induced by other stimulus besides face, such as car, house, furniture, words [30], [31], [32]. In the linguistic field, studies on N160 or N170 component in the visual cognition mechanism of words were primarily focused on the language family of alphabets. Many have discovered that words would induce more N160 or N170 reactions in the left occipital lobe compared with other stimulus like human faces, object drawings, or character strings [33], [34], [35], [36]. While in the working memory field, McEvoy's study [23] found both spatial working memory task and verbal working memory task can induce N160 component in parietal and occipital, and the N160 component can be interpreted as being relevant with perceptual speed and visual attention allocation.

**Figure 3:**
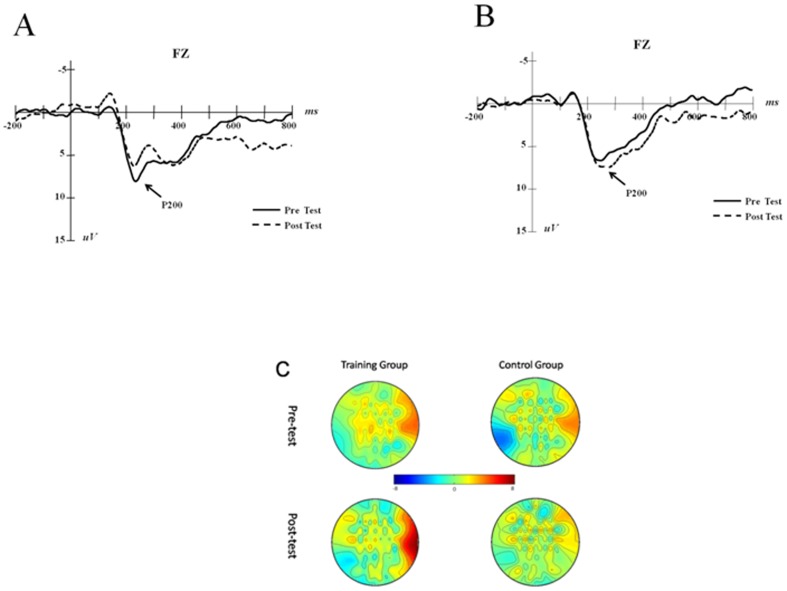
P200 (FZ) trends in the training and control groups. A is P200 changes between the pre- and post-test in the training group, and B is the results of the control group. Scalp topographies of the two groups on P200 are demonstrated on C.

In the individual P200 component analysis, we used the post-test peak value minus the pre-test peak value to obtain the additive values, and we calculated the two groups' average additive values (See Figure 3). The independent sample t-test showed that the additive value of P200 of the training group is significantly lower than that of the control group (*t*(21) = −3.020, *p* = 0.007, *d* = 1.26).

These results showed that the updating training effect presents as not only an increase in P300 amplitude but also a decrease in P200 in the frontal area. The P200 evoked from the frontal area is considered to reflect the inhibition of irrelevant information and the ability to attend to the target stimulus [23], [37]. Additionally, in working memory aging studies, the P200 in the frontal area is significantly lower in old subjects than in the young subjects [23]. Engle et al. proposed a working memory executive attention model [38].This model demonstrated that executive attention is a domain-general and limited executive processing or an underlying mechanism that, when faced with interfering or distracting stimuli, maintains attention on task-relevant information [10]. In terms of working memory updating ability, its main function is to monitor input information and to replace old information that is irrelevant to the ongoing task with new information that is relevant to the ongoing task [22], [25].Thus, the decreased P200 in the frontal area indicated that the training improved the crucial factor in working memory, which is executive attention.

**Figure 4:**
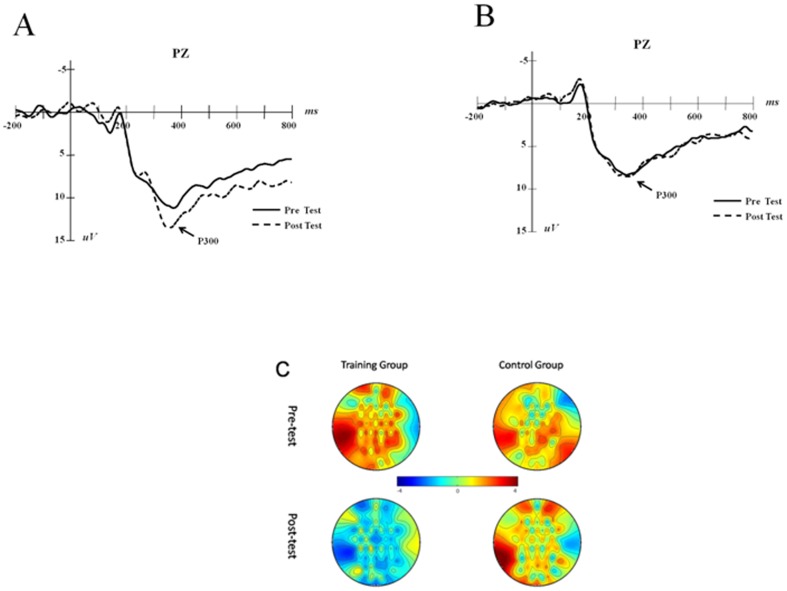
P300 (PZ) trends in the training and control groups. A is P300 changes between the pre- and post-test in the training group, and B is the results of the control group. Scalp topographies of the two groups on P300 are demonstrated on C.

For individual P300 component analysis, we used the post-test peak value minus the pre-test peak value to obtain the additive values, and we calculated the two groups' average additive values (See Figure 4). The independent sample t-test showed that the additive value of P300 of the training group is significantly higher than that of the control group (*t*(21) = 2.104, *p* = 0.048, *d* = 0.88).

P300 has been well established as a valid index for updating working memory. According to the context updating model, when information is presented, brains will respond and fit the new information into current knowledge, forming new representations to replace the old ones for future experiences, depending on the relevance of the new information to the subject. When the environment is altered continuously, the current knowledge needs to be adjusted to varying degrees. This process may generate the P300 peak [28], [39], [40]. Previous studies have shown that individuals with high cognitive ability tend to have higher P300 amplitudes [28].Additionally, evidence from aging research has demonstrated that, as aging progresses, the P300 amplitude is reduced [23]. Our results showed that the P300 amplitude increased after the training, which indicates that the training could also improve the updating function in working memory representation.

We made the correlation analysis about the increment of accuracy, RT, and additive value of N160, P200, and P300. Results are displayed on table 3. It seems that there is no significant correlation among all behavioral and ERP component changes. However, the increment of accuracy and additive value of P300 have trend to be positively correlated.

**Table 3:** Correlations of Increment in accuracy, reaction time and additive value in N160, P200, P300.

		P300	P200	N160(P7)	N160(P8)
Increment in accuracy	*r*	.55	.01	.11	.36
	*p*	.07	.98	.74	.25
Increment in reaction time	*r*	.07	.02	.44	.37
	*p*	.82	.94	.15	.24

Compared with previous studies [16], [20], [21], our study proved that working memory training first enhanced the stimulus recognition ability of individuals at the visual recognition stage and then strengthened the capacity to inhibit irrelevant information and attend to current target stimuli, in turn improving the updating ability in working memory representation. During these processes, multiple brain areas from the parietal to frontal and back to the parietal cortex participated and cooperated. Our study contributes to the working memory plasticity research. Future studies will combine ERP and fMRI techniques to record the time course and locate the brain activity in certain areas of working memory updating processing.
